# Severity over source: Understanding transgressors' motive attributions of punishment

**DOI:** 10.1111/bjso.70118

**Published:** 2026-08-28

**Authors:** Mathias Twardawski, Dorothee Mischkowski, Stefanie Hechler

**Affiliations:** ^1^ Department of Psychology Ludwig‐Maximilians‐Universität München Munich Germany; ^2^ Max Planck Institute for Research on Collective Goods Bonn Germany; ^3^ Social, Economic and Organisational Psychology Unit, Institute of Psychology Leiden University Leiden Netherlands; ^4^ Einstein Research Unit ‘Coping with Affective Polarization’ Freie Universität Berlin and Humboldt‐Universität zu Berlin Berlin Germany

**Keywords:** motive attribution, prosociality, reconciliation, second‐party punishment, third‐party punishment

## Abstract

Punishment conveys social information to transgressors, who attribute motives to punishers. Drawing on the motive‐attribution framework, we examined whether transgressors' attributions to *competitive* (i.e. harming transgressors), *individualistic* (i.e. managing one's own interests) and *prosocial* (i.e. healing relationships) motives differ depending on whether punishment is administered by victims (second‐party punishment) or uninvolved observers (third‐party punishment), and how these attributions relate to downstream affective, relational and behavioural outcomes. In an incentivized Stealing Game, participants could steal valuable points and were punished either by the victim or an observer. Subsequently, transgressors reported their motive attributions, affect and willingness to reconcile and completed a measure of prosocial behaviour towards an unrelated other. Contrary to predictions, motive attributions did not systematically differ between second‐ and third‐party punishment, except that third‐party punishment was more strongly attributed to reputational concerns (i.e. a subdimension of individualistic motive attribution). Punishment severity emerged as an important predictor, with harsher sanctions linked to stronger competitive and individualistic attributions. Competitive motive attribution, in turn, was associated with higher negative affect and lower positive affect, willingness to reconcile and prosocial behaviour. Overall, transgressors' responses to punishment were less clearly shaped by who punished and more closely linked to punishment severity and motive attribution.

## INTRODUCTION

Punishment typically involves the deprivation of positive stimuli, such as freedom, rights or money, or the infliction of negative stimuli—in extreme cases, even harm to one's physical condition (e.g. Foucault, 1977). Recent approaches, however, suggest that punishment likely not only functions as a simple retributive mechanism (i.e. to get back at the transgressor) or a deterrence mechanism (i.e. to teach transgressors to avoid certain types of behaviour). Rather, punishment should be understood as a social interaction that conveys messages to transgressors (Cushman et al., 2022). More precisely, punishment carries important communicative values that go beyond merely treating transgressors negatively: It communicates that the transgressor's behaviour was wrong and may thereby instigate social learning about how individuals behave appropriately in the eyes of the punishers.

However, the message that punishment conveys depends not only on the sender (i.e. the punisher), but also on the punished individual who receives and interprets the message. Gollwitzer and Okimoto (2021) have recently emphasized this idea in the *motive‐attribution framework*. Building on attribution theory (Kelley, 1973), they argue that transgressors try to make sense of their punishment by making attributions about *why* someone chose to punish them. These attributions subsequently influence their emotions, attitudes and responses (e.g. towards the punisher; Pronin, 2008). Along these lines, recent research has demonstrated that, when authorities communicate their punishment respectfully (vs. disrespectfully), transgressors attribute it more strongly to benevolent versus malevolent motives and, consequently, show increased prosocial attitudes and behaviour (de Vel‐Palumbo et al., 2023). We build upon this theorizing and suggest that transgressors’ attributions of punishment not only depend on the act of punishment itself, that is, *how* the message is communicated, but also on *who* punishes, that is, *the source* of the message and their role in the initial transgression.

Based on the conceptual structure of Social Value Orientation (SVO; Van Lange, 1999), the motive‐attribution framework suggests that punishment can be attributed to *competitive* (i.e. harm‐oriented), *individualistic* (i.e. self‐oriented) and/or *prosocial* (i.e. relationship‐ or other‐oriented) motives (Gollwitzer & Okimoto, 2021).^1^ Competitive motives are geared towards harming transgressors and making them suffer for the harm they caused. This is closely related to the notion that people are retributivists and punish transgressors to rebalance the scales of justice (e.g. Aharoni & Fridlund, 2012; Carlsmith & Darley, 2008). In turn, individualistic motives aim to manage the punisher's own circumstances, such as (re)gaining social status or a sense of personal control and obtaining reputational benefits (Bies & Tripp, 1996; Raihani & Bshary, 2015a; Stillwell et al., 2008). It may come with some surprise that punishment can also be attributed to prosocial motives of the punisher: By delivering a message about their inappropriate behaviour to transgressors, punishment may aim to heal relationships (e.g. in marital transgressions; Fitness & Peterson, 2008), help the transgressor become a better person (e.g. punishment by teachers in schools; Reyna & Weiner, 2001; Twardawski et al., 2020) or re‐integrate the transgressor into the community (e.g. through ‘reintegrative shaming’; Braithwaite, 1989). Importantly, the motive‐attribution framework proposes that the downstream consequences of punishment crucially depend on how the transgressor interprets the punishment (Gollwitzer & Okimoto, 2021). More specifically, punishment may be more likely to yield positive consequences in transgressors (e.g. acceptance by the transgressor), the more they attribute it to prosocial motives; whereas punishment may be more likely to yield negative consequences (e.g. withdrawal of the transgressor), the more they attribute it to competitive or individualistic motives.

In the present research, we aim to investigate whether the source of the punishment influences the transgressor's attribution regarding the underlying punishment motives. Punishment may be a direct response from victims of the initial transgression, such as rejecting unequal offers in the ultimatum game (e.g. Güth et al., 1982; Mendoza et al., 2014). This is referred to as *second‐party punishment*. Sometimes, however, punishment is imposed by uninvolved observers who are not personally affected by the transgression (see e.g. Fehr & Gächter, 2002). This is referred to as *third‐party punishment* (Boyd et al., 2003; Fehr & Fischbacher, 2004a).

In general, second‐ and third‐party punishment share important similarities. For both second (i.e. victims) and third parties (i.e. observers) alike, engaging in punishment requires investing their own resources to impact a transgressor's conditions, such as their monetary outcomes (e.g. Fehr & Gächter, 2002). Additionally, direct comparisons of second‐ and third‐party punishment reveal that both are directed at individuals who harbour harmful intentions or produce harmful consequences or inequality (Gummerum & Chu, 2014; Hechler & Kessler, 2022; Leibbrandt & López‐Pérez, 2012). However, there are also important differences between second‐ and third‐party punishment. For example, second parties tend to choose harsher punishments (Civai et al., 2019; Crockett et al., 2014; Hechler & Kessler, 2022) and focus more on the reciprocity of harm than third parties (Leibbrandt & López‐Pérez, 2012). Moreover, second parties report more general anger after transgressions; whereas third parties report feeling more ‘moral outrage’—yet, both emotions correlate positively with punishment severity (Hartsough et al., 2020; Hechler & Kessler, 2018).

Importantly, even though the consequences of punishment for transgressors may in principle be similar (i.e. the amount of harm inflicted on the transgressor), people perceive and evaluate the punishment differently depending on the role of the punisher in the initial interpersonal transgression, namely, whether they are victims or observers (Martin et al., 2019; Mifune et al., 2020). Moreover, third‐party punishment is more likely to lead to people adopting prosocial norms (i.e. making a fair distribution in a Dictator Game) compared to second‐party punishment (Chen et al., 2020). This and other previous research, however, has predominantly focused on how *observers* evaluate and respond to second‐ versus third‐party punishment (Eriksson et al., 2016; Gordon et al., 2014; Kupfer & Tybur, 2023). In the present study, we extend this approach and propose that the source of punishment also produces differential effects *on transgressors* because they attribute different motives to different punishers. Thus, we aim to investigate the differences in transgressors' motive attributions to punishment by victims (i.e. second‐party punishment) and by observers (i.e. third‐party punishment).

Research on the transgressors' perception of second‐ versus third‐party punishment is, to the best of our knowledge, strikingly scarce. Previous research, however, suggests that transgressors may attribute motives to punishers through introspection—asking themselves what they would aim to achieve when punishing as either the victim or an observing third party (Davis et al., 1996). Building on this reasoning, we derive hypotheses about transgressors' attributions of second‐ and third‐party punishment based on both the results of research investigating the observers' perspective (e.g. Chen et al., 2020; Mifune et al., 2020) and the motives that previous research associated with victims and observers. In what follows, we outline the hypotheses for the attribution of prosocial, competitive and individualistic motives separately.

Hypotheses regarding the attribution of *prosocial motives* to punishment can be derived from literature that describes punishment as a tool to facilitate social norms (Fehr & Fischbacher, 2004b). Punishment may follow a prosocial orientation as it effectively promotes compliance with cooperative norms (e.g. Fehr & Gächter, 2002) and thereby maximizes joint gains for all parties involved in subsequent interactions (Balliet et al., 2011). This aligns with the notion of altruistic punishment (Fehr & Gächter, 2002), implying that costly (third‐party) punishment may—at least in part—be driven by prosocial motives (note, however, that the concept of altruistic punishment has lately received strong criticism; e.g. Brethel‐Haurwitz et al., 2016). Recent findings further suggest that third‐party punishment increases cooperation more strongly than second‐party punishment, as it more clearly communicates consensual cooperative norms (Chen et al., 2020; Zhou et al., 2017). Moreover, observers tend to perceive third‐party punishers as more trustworthy and cooperative (Kupfer & Tybur, 2023) and as generally more likable than third parties who refrain from punishing norm violators (Gordon et al., 2014). By contrast, second‐party punishers are perceived as less trustworthy and are judged to have a worse character overall (Eriksson et al., 2016). Again, although these studies focus on the perceptions of outside observers, they suggest that third‐party punishment is associated with positive characteristics (e.g. trustworthiness). We therefore hypothesize that transgressors will attribute prosocial motives more strongly to third‐party than to second‐party punishers.

Regarding the attribution of *competitive motives* to second‐ versus third‐party punishment, punishment may be perceived as aiming at making the transgressor suffer (Gollwitzer & Denzler, 2009). This aligns with the notion that people are retributivists, often punishing according to the principle of ‘an eye for an eye’, seeking to make the transgressor suffer as a repayment for the harm caused by the transgression (Carlsmith & Darley, 2008). Importantly, this punishment motive seems to be stronger among victims compared to uninvolved third parties (Crockett et al., 2014; Leibbrandt & López‐Pérez, 2012). Moreover, observers tend to view second‐party punishers more strongly as retaliatory or vengeful figures than third‐party punishers (Raihani & Bshary, 2015b, 2019), supporting the notion that transgressors may attribute second‐party punishment more strongly to competitive motives than third‐party punishment.

As for the attribution of *individualistic motives* to punishment, competing hypotheses can be formulated: On the one hand, the desire to restore personal status and power may drive second‐party punishers as victims were threatened by the experienced victimization (Wenzel et al., 2008). Correspondingly, second‐party punishment (but less so third‐party punishment) aims at experiencing and regaining power (Strelan et al., 2020) and self‐esteem (Zdaniuk & Bobocel, 2012). The motive‐attribution framework associates this with individualistic motives. Therefore, transgressors may attribute such motives more strongly to second‐party than third‐party punishers. On the other hand, individualistic motives may also drive third‐party punishment—but on a different (sub)dimension. That is, third‐party punishment (but less so second‐party punishment) has been found to enhance reputational benefits (Raihani & Bshary, 2015a), signalling alliances and helping to maintain beneficial relationships (DeScioli & Kurzban, 2013). Consequently, transgressors may assume that concerns for one's reputation and social embeddedness motivate third‐party punishment and, thus, more strongly attribute it to individualistic motives than second‐party punishment. In sum, transgressors may attribute both second‐ and third‐party punishment to individualistic motives, with a potential qualitative difference between these motive attributions (i.e. on different dimensions, as suggested by competing theoretical accounts).

### The Present Research

Recently, the motive‐attribution framework suggested that the effect of punishment on transgressors is conditional on the degree to which they attribute the punishment to competitive, individualistic and prosocial motives of the punisher (Gollwitzer & Okimoto, 2021). Herein, we suggest that this attribution may depend on the source of the punishment, that is, whether the victims of the transgression (i.e. second‐party punishment) or uninvolved observers (i.e. third‐party punishment) carry out the punishment, even when the severity of punishment is identical.

Only recently has research compared the effects of second‐ versus third‐party punishment on uninvolved observers, showing that third‐party punishment is more capable of instigating subsequent norm compliance of these observers (Chen et al., 2020). Crucially, research examining the transgressors' perception and subsequent reaction to punishment is still lacking, although this may be of high relevance for any post‐conflict reconciliation processes (Shnabel & Nadler, 2008). In the present experiment, we therefore aim to test the following hypotheses:

1. Transgressors attribute third‐party punishment more strongly to prosocial motives than second‐party punishment.
2. Transgressors attribute second‐party punishment more strongly to competitive motives than to third‐party punishment.
3. Transgressors' attribution of second‐party punishment and third‐party punishment to individualistic motives differs on two (sub)dimensions of individualistic motives:
  - They attribute second‐party punishment more strongly to power‐restoration and self‐esteem regulation than third‐party punishment.
  - They attribute third‐party punishment more strongly to reputational concerns than to second‐party punishment.

A vital prediction of the motive‐attribution framework is that punishment is more likely to yield positive consequences in transgressors when they attribute it to prosocial motives of the punisher. Specifically, such attributions should decrease transgressors' negative affect associated with being punished, while increasing positive affect and the transgressors' willingness to reconcile with the punisher (Eadeh et al., 2017; Gollwitzer & Okimoto, 2021). By contrast, punishment is more likely to yield negative consequences in transgressors (e.g. rejection or hostility) when they attribute it to competitive or individualistic motives of the punisher. Ultimately, these motive attributions are also expected to shape transgressors' post‐punishment behaviour, particularly their prosocial behaviour (i.e. resource allocations; Van Lange, 1999). Specifically, attributing punishment to prosocial motives should correlate positively with subsequent prosocial tendencies; whereas attributing it to competitive or individualistic motives should correlate negatively with such tendencies (de Vel‐Palumbo et al., 2023).

Based on these considerations, we propose the following predictions:

4 Punishment attributions predict transgressors' positive affect, negative affect, willingness to reconcile with the punisher and post‐punishment prosocial behaviour.
  4.1 Willingness to reconcile with the punisher (a) increases with higher attributions to prosocial motives and decreases with higher attributions to (b) competitive and (c) individualistic motives. These effects (a, b and c) are expected to occur independently, meaning that any one of them might occur without the others.
  4.2 Positive affect (a) increases with higher attributions to prosocial motives and decreases with higher attributions to (b) competitive and (c) individualistic motives. These effects (a, b and c) are expected to occur independently, meaning that any one of them might occur without the others.
  4.3 Negative affect (a) decreases with higher attributions to prosocial motives and increases with higher attributions to (b) competitive and (c) individualistic motives. These effects (a, b and c) are expected to occur independently, meaning that any one of them might occur without the others.
  4.4 Prosocial behaviour (a) increases with higher attributions to prosocial motives and decreases with higher attributions to (b) competitive and (c) individualistic motives. These effects (a, b and c) are expected to occur independently, meaning that any one of them might occur without the others.

To test these predictions, we conducted an experiment employing the Stealing Game as an experimental paradigm (e.g. Harbaugh et al., 2013). In this paradigm, participants had the opportunity to commit a transgression by stealing money from another participant, knowing there was a chance their behaviour might be detected and punished. Punishment was then administered either by the victim (i.e. the person from whom the money was taken; second‐party punishment) or by an uninvolved observer (i.e. third‐party punishment). The role of the (potential) punisher in the initial transgression (victim vs. observer) was experimentally manipulated. Following the punishment, we measured the key dependent variables, that is, the transgressors' attributions of punishment to prosocial, competitive and individualistic motives. To assess potential downstream consequences of motive attributions, we measured transgressors' negative and positive affect, as well as their willingness to reconcile with the punisher. Additionally, to evaluate whether punishment (and its interpretation) is associated with transgressors' subsequent prosociality, participants completed the SVO Slider Measure (Murphy et al., 2011), which assesses prosocial behaviour by means of resource allocations between themselves and another person with whom they did not interact before.

## METHODS

### Transparency and openness

We report how we determined our sample sizes, all data exclusions (if any), all manipulations and all measures (Simmons et al., 2012). This Registered Report was pre‐registered prior to the start of data collection: https://osf.io/dk2ub/. The pre‐registration, materials, data, codebook and analysis script of the study are available on the Open Science Framework (OSF): https://osf.io/w7abx/. This study was approved by the Ethics Council of the Max Planck Society within the framework of the Generalized Approval of Experiments Following the Protocol that is Standard in Experimental Economics (approval no. 2018_3 – Renewal 2024_28).

### Sample

To determine the required sample size, we conducted an a priori power analysis using G*Power (Faul et al., 2009) to test our central prediction—that motive attributions differ between conditions (i.e. second‐ vs. third‐party punishment)—through three separate but identical tests corresponding to Hypotheses 1–3. Assuming a small‐to‐medium‐sized effect (*f* ^2^ = .07) in a linear multiple regression (fixed model, *R* ^2^ increase with total number of predictors = 4, number of tested predictors = 1, α = .05 and 1‐β = .90), this analysis yielded a required sample size of *N* = 153 participants in the role of a punished transgressor (who completed all relevant outcome measures).

We pre‐registered collecting data until reaching a sample size of *N* = 153 participants in the role of the transgressor who are eligible for our analyses (i.e. after data exclusions; see below). Participants in the other roles of the Stealing Game (i.e. victims and third parties) were additionally recruited to enable real interactions in accordance with behavioural economic standards, but their responses are not part of the main hypothesis tests of this study. Given that punishment rates were expected to be higher in the second‐party punishment condition (compared to the third‐party punishment condition), we anticipated that more eligible participants would come from that condition. To ensure sufficient power for between‐condition comparisons, we defined a secondary stopping rule: a minimum of 76 eligible participants in the third‐party punishment condition.

Participants were recruited via the decision lab's local participant pool. This pool primarily includes university students, although non‐student volunteers may also be included. The experiment adhered to behavioural economic standards, that is, all decisions had real monetary consequences, and no deception was used. Specifically, a random selection between the outcomes of either the Stealing Game or the SVO Slider Measure determined participants' payments. The average value regarding the outcome of both paradigms was approximately equal, with *M* = 3.50 € for the Stealing Game and *M* = 3.31 € for the SVO. Consequently, participants received variable payments based on their decisions in the study (*M* = 3.43 €, *Min* = 0.40 €, *Max* = 6.80 €), in addition to a fixed flat fee of 2.00 €. Participation took approximately 30 min. Participants were provided with all necessary information to give their informed consent, in accordance with the ethical guidelines of the German Psychological Society.

Overall, 604 participants started the study and 496 completed it (with 251 in the role of the potential transgressor). As pre‐registered, we excluded 11 participants from data analyses who failed to correctly answer three comprehension‐check questions about the experimental paradigm after three attempts (i.e. the Stealing Game).^2^ Moreover, we excluded 46 participants who decided not to steal any points, and an additional 20 participants who were not detected or punished by either victims or third parties. After data exclusions, the sample consisted of *N* = 419 participants, including 174 punished transgressors. Of these, 96 were detected and punished by their victim and 78 were detected and punished by a third party.

The final sample of 174 transgressors had a mean age of *M* = 28.94 years (*SD* = 10.35; range: 18–74). The sample included 57 men, 114 women, 2 non‐binary participants and 1 participant who did not report their gender.

### Design and materials

The experiment employed a between‐subjects design, in which the potential punisher in the Stealing Game was manipulated as the victim of the transgression (second‐party punishment) or an uninvolved observer (third‐party punishment). Detailed instructions for the Stealing Game, as well as items of all scales, are available on the OSF.

### Stealing Game

In the instructions of the Stealing Game (Harbaugh et al., 2013), participants were informed that they would interact with two anonymous other participants. Furthermore, participants were told that the interaction would involve the distribution of points that were later converted into real money.

In the interaction, participants were randomly assigned to one of three roles: Person A (i.e. the potential transgressor), Person B (i.e. the potential victim) or Person C (i.e. the observer). As our research question focused on transgressors' motive attributions, the role of Person A was most central. All information on the procedure and dependent variables provided below pertains to participants in this role.

As an initial endowment, Person A received 25 points, Person B received 38 points, and Person C received 23 points. To echo stealing behaviour in the real world, Person A was not informed about the initial endowments of Person B and Person C, but only about the possible amount that could be stolen (between 0 and 15 points).

**FIGURE 1:**
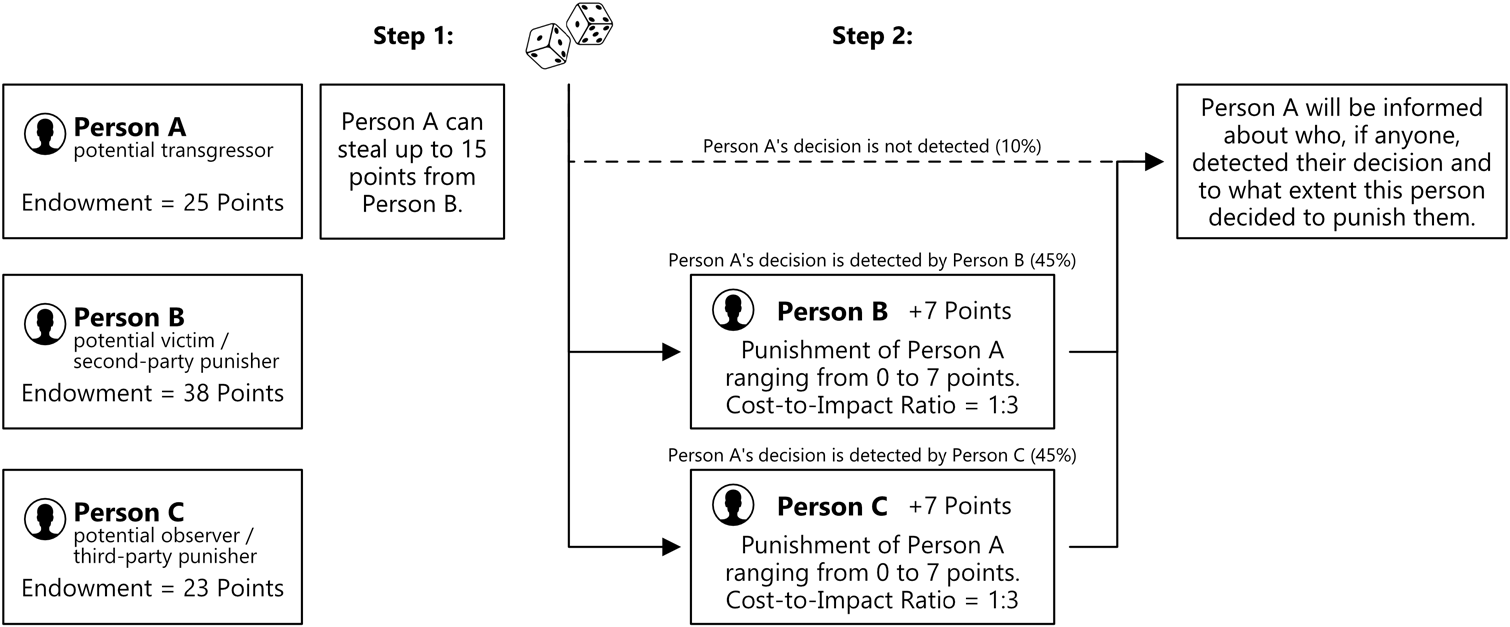
Graphical illustration of the Stealing Game.

The interaction itself consisted of two steps: First, Person A could take up to 15 points from Person B (this decision was framed as the opportunity to ‘take away’ points). Second, there was a chance that Person A's decision was detected and punished, either by Person B (45%) or by Person C (45%). To reduce the risk of floor effects in stealing behaviour, Person A's decision remained undetected in 10% of cases (in this case, participants in the role of Person A directly continued with the SVO Slider Measure after making their decision in the Stealing Game). In the remaining 90% of cases, the person who detected the behaviour was either Person B or Person C (randomly assigned). These potential punishers received an additional endowment of 7 points and could invest between 0 and 7 points to punish Person A. Each point they invested was lost to them (i.e. costly punishment), but Person A's payment was reduced by 3 points (i.e. a cost‐to‐impact ratio of 1:3). Person A was informed about whether their decision was detected and, if so, by whom and to what extent this person decided to punish them.^3^ The procedure of the Stealing Game is illustrated in Figure 1.

To secure sufficient punishment in the Stealing Game, we conducted a pilot study with *N* = 60 participants in line with behavioural economic standards (i.e. incentivized decision‐making, no deception). Of the 30 individuals who were assigned to the role of Person A, 86.7% (*n* = 26) engaged in stealing, with an average amount of 10.23 points being stolen (*SD* = 5.59). This result suggested that the task successfully elicits transgressive behaviour. In addition, we tested the punishment behaviour of participants assigned to the roles of Person B and Person C. On average, participants imposed stronger punishments in the role of Person B (*M* = 2.73, *SD* = 1.34) than in the role of Person C (*M* = 2.06, *SD* = 1.67). Only 7.1% of participants in role B and 21.4% in role C entirely refrained from punishing. Moreover, punishment increased with the amount stolen (overall: *r* = .61; for Person B only: *r* = .71; for Person C only: *r* = .51). Based on these results, the task suggested to yield sufficient behavioural variance for the planned analyses.

### Comprehension and attention checks

To ensure sufficient understanding of the Stealing Game, participants answered three comprehension‐check questions to continue with the experiment after instructions; if answered incorrectly, they had to review the instructions and attempt the questions again. After three incorrect attempts, participants continued with the experiment but their data were not analysed.

To validate awareness of the experimental manipulation (i.e. whether participants were aware who detected and potentially punished them), participants in the role of a detected Person A were asked to indicate who detected their decision (Person B, Person C or nobody) as an attention check. If they answered this question incorrectly, they were asked to read the corresponding information again before proceeding.

### Motive attribution

Participants indicated their motive attributions using a self‐report measure developed in accordance with the motive‐attribution framework (de Vel‐Palumbo et al., 2023; Twardawski et al., 2023). We adapted this measure to fit the context of the experiment (i.e. an interaction in an economic game). The final instrument consisted of 16 items. Each motive was measured with four items. One example item for prosocial motive attribution is ‘Person B (second‐party punishment condition) / Person C (third‐party punishment condition) reacted in this way to promote harmony within the group’; and for competitive motive attribution ‘[…] to make me feel bad’. Attribution to individualistic motives was divided into (a) four items related to power‐restoration and self‐esteem regulation (e.g. ‘[…] to boost his/her own self‐worth’) and (b) four items related to reputational concerns, such as being seen in a positive light or as a person with integrity (e.g. ‘[…] to improve their own reputation’). Results of the pre‐registered exploratory factor analysis (EFA) and internal consistencies of the derived scales are reported in the results section.

### Positive and negative affect

To measure positive and negative affect, participants indicated how they felt after being punished (six items for positive and six for negative affect) (Eadeh et al., 2017). Example items for positive affect include ‘I feel happy’ and ‘I feel satisfied’. Example items for negative affect include ‘I feel sad’ and ‘I feel anxious’. Scale scores for positive and negative affect were computed separately by averaging the corresponding items within each subscale. Internal consistencies were high, with α = .91 for positive affect and α  = .85 for negative affect.

### Willingness to reconcile

Participants' willingness to reconcile with the punisher was measured using 10 items from Twardawski et al. (2023). One example item is ‘I suppose I and Person B (second‐party punishment condition)/Person C (third‐party punishment condition) could have a positive relationship in the future’. A composite reconciliation score was created by averaging all items. Internal consistency was high (α = .90).

All response scales (i.e. for motive attributions, affect and willingness to reconcile) ranged from 1 = ‘strongly disagree’ to 6 = ‘strongly agree’.

### Social value orientation

**FIGURE 2:**
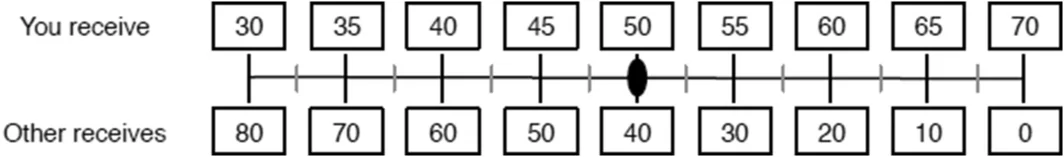
Graphical illustration of an example in the SVO Slider Measure.

Participants' prosocial behaviour was assessed using the 15‐item version of the SVO Slider Measure (Murphy et al., 2011), in which participants distribute monetary units between themselves and another person. To do so, participants were matched with a random other participant with whom they had not interacted before to avoid reciprocal concerns. The items involve a trade‐off between the maximization of one's own welfare and the other person's welfare. A graphical illustration of an example item can be found in Figure 2.

In this example, selfishness (maximizing one's own outcome) is reflected in the choice of the last option (70 to self, 0 to the other), whereas prosociality is reflected in the first option (30 to self, 80 to the other). Besides the differentiation between proself and prosocial, the measure also distinguishes between an altruistic and competitive value orientation. The former aims solely to maximize the outcome of the other person and is thus an extreme form of prosociality. At the other end of the spectrum, a competitive value orientation is reflected in maximizing the difference between one's own and the other's outcome. These four categories are assessed within the first six (i.e. primary) items of the SVO Slider Measure, yielding not only the categorical classification, but also a continuous SVO angle that increases with increasing prosociality (this angle was used in all our analyses). For exploratory purposes, we further included nine secondary items that differentiate among prosocial motives (not pertinent for the present research). Participants were incentivized based on one randomly selected decision from the SVO Slider Measure. Specifically, each participant was paired with another individual, and one of the decisions made by either person determined the outcomes for both.

The SVO Slider Measure is a suitable measure for the present purpose for various reasons. First, it is a theoretically grounded and interpretable proxy for potential shifts in interpersonal orientation following punishment attributions (Gollwitzer & Okimoto, 2021). Second, the measure allows for assessing more than mere behavioural suppression. Beyond examining whether transgressors refrain from repeating a specific act (e.g. stealing), we were interested in whether attribution of punishment motives can foster broader prosocial engagement. Third, the measure has repeatedly demonstrated satisfactory psychometric properties and good construct validity (Hilbig et al., 2014; Mischkowski et al., 2019; Murphy et al., 2011). Fourth, it has been shown to respond to contextual and experimental manipulations (e.g. Messick & McClintock, 1968), making it suitable for detecting attribution‐related shifts in social orientation. Finally, using the SVO Slider Measure instead of a second round of the Stealing Game may reduce the likelihood of transgressors using it to morally license their prior behaviour and not as a consequence of the punishment (however, see mixed evidence for the role of contextual similarity on moral licensing; Blanken et al., 2015; Martuza & Kim, 2025). A conceptually distinct task, like the SVO Slider Measure, mitigates the risk of confounding interpretations while capturing potential meaningful variation in social preferences and their relation to the experimental conditions and attributions.

### Demographics and exploratory variable

Participants reported their age and gender. As an exploratory variable, we assessed participants' awareness of their wrongdoing and the perceived immorality of their own behaviour in the Stealing Game (in the role of Person A) with three items (e.g. ‘I feel my actions were unethical’, *α* = .85) on a scale from 1 = ‘strongly disagree’ to 6 = ‘strongly agree’.

### Procedure

**FIGURE 3:**
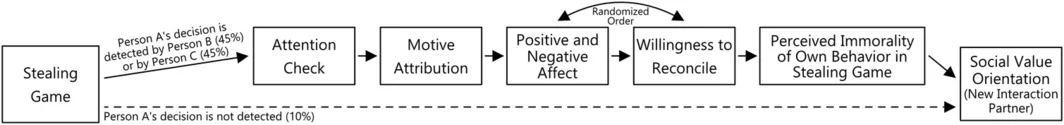
Procedure of the study (for participants in the role of Person A).

The experiment was programmed and conducted online using the software *oTree* (Chen et al., 2016). After providing informed consent, participants were randomly allocated to a role in the Stealing Game and made their decisions according to their role. For participants in the role of Person A, the study continued with the attention check, followed by the motive‐attribution questionnaire (except for participants in the role of Person A who were not detected in the Stealing Game, who continued with the SVO Slider Measure). Positive and negative affect, as well as willingness to reconcile, were then assessed in randomized order. Participants subsequently indicated their awareness of their wrongdoing and the perceived immorality of their own behaviour in the Stealing Game (exploratory measure). The experiment concluded with the SVO Slider Measure. Finally, participants provided demographic information before they were thanked, informed about their final payout and debriefed. An overview of the study procedure for participants in the role of Person A is depicted in Figure 3. Participants in the roles of Person B/Person C also answered several questionnaires for exploratory purposes and completed the SVO Slider Measure. Participants' final payment was based on the results of the SVO Slider Measure in 50% of cases, while the outcomes of the Stealing Game determined payment in the remaining 50%. Given that the experiment was conducted online, all participants received their payments via bank transfer within two weeks after data collection.^4^

## RESULTS

### Dimensionality of the motive‐attribution scale

As pre‐registered, we first examined the dimensionality of the motive‐attribution scale and evaluated item loadings before constructing the final scales for analysis. The parallel analysis indicated a three‐factor solution. We then conducted an exploratory factor analysis (EFA) using minimum residuals as the extraction method and oblimin rotation, as factors were expected to correlate. Detailed results of the EFA are provided in the supplementary materials on the OSF.

Overall, the EFA indicated some deviations from the theoretically suggested structure. One item originally designed to measure prosocial motive attribution and five items originally designed to measure (the two subdimensions of) individualistic motive attribution did not load sufficiently on the expected factor (<.30) or showed substantial cross‐loadings (>.30 on more than one factor). As pre‐registered, these items were excluded from all main analyses. The resulting scales demonstrated acceptable internal consistencies: prosocial motive attribution (α = .73), individualistic motive attribution (α = .71) and competitive motive attribution (α = .90). Notably, the resulting individualistic motive scale contained only one item that was originally formulated to capture reputational concerns and two items originally formulated to capture power‐restoration/self‐esteem concerns.

The three‐factor solution derived following the pre‐registered EFA does not allow distinguishing between the two theoretically important subdimensions of individualistic motive attribution: power‐restoration/self‐esteem versus reputational concerns. Yet, to test Hypotheses 3a and 3b, we additionally constructed these two subscales using the respective four items each based on theoretical considerations only (i.e. without excluding any items based on item loadings). Both subscales demonstrated satisfactory internal consistencies (power‐restoration/self‐esteem: α = .70; reputational concerns: α = .72).

### Stealing and punishment behaviour

194 participants engaged in stealing as Person A. The average amount stolen was 11.31 points (i.e. 75.4% of the maximum stealing possible; *SD* = 4.14 points, range = 2–15). This indicates both substantial stealing and variability in the amount stolen.

Across both roles (second and third parties) and all punishment decisions, punishers invested on average 2.22 points (31.7% of the maximum punishment, *SD* = 1.78 points) to sanction transgressors. Beyond overall punishment levels, we compared punishment imposed by second and third parties using a paired‐samples *t*‐test. Second‐party punishment (*M* = 2.40, *SD* = 1.80) was significantly higher than third‐party punishment (*M* = 2.05, *SD* = 1.93), *t*(244) = 4.83, *p* < .001, *d_z_* = 0.31. Moreover, the amount stolen was positively associated with punishment severity overall, *r* = .42, *p* < .001. This association was slightly stronger for second‐party punishment, *r* = .48, *p* < .001, than for third‐party punishment, *r* = .37, *p* < .001.

### Descriptive statistics

We examined descriptive statistics and bivariate correlations among all core variables (see Table 1).^5^ Prosocial, competitive and individualistic motive attributions were intercorrelated, although the magnitudes differed substantially across dimensions. Consistent with the motive‐attribution framework, prosocial attribution showed a weak but significant positive correlation with willingness to reconcile, whereas its correlations with affect and prosocial behaviour (i.e. the SVO angle) were non‐significant and close to zero. By contrast, competitive attribution was strongly positively correlated with negative affect and strongly negatively correlated with positive affect, willingness to reconcile and (to a weaker extent) prosocial behaviour. The overall individualistic attribution scale showed a similar pattern (yet, correlations were weaker): a positive correlation with negative affect and a negative correlation with positive affect. Its weak negative correlations with willingness to reconcile and prosocial behaviour were both non‐significant.

**TABLE 1:** Means, standard deviations and correlations of all measured variables.

Variable	*M*	*SD*	1	2	3	4	5	6	7	8	9	10	11	12
1. Prosocial attribution	3.14	1.29	.73											
2. Competitive attribution	2.75	1.59	.12	.90										
3. Individualistic attribution	3.44	1.23	.48**	.37**	.71									
4. Ind. reputation attribution	3.09	1.21	.57**	.11	.68**	.72								
5. Ind. power/self‐esteem Attr.	3.61	1.15	.30**	.54**	.83**	.44**	.70							
6. Positive affect	2.94	1.36	.00	−.60**	−.26**	.07	−.39**	.91						
7. Negative affect	2.02	1.07	.02	.47**	.23**	.01	.34**	−.43**	.85					
8. Willingness to reconcile	3.90	1.07	.17*	−.45**	−.12	.17*	−.28**	.45**	−.51**	.90				
9. Immorality of own behaviour	2.73	1.41	.31**	.11	.15*	.21**	.11	.06	.08	.08	.85			
10. Punishment severity	3.17	2.76	.02	.62**	.28**	−.05	.39**	−.65**	.38**	−.41**	−.05	–		
11. Amount stolen	11.29	4.09	−.07	.27**	.01	−.09	.09	−.22**	.17*	−.19*	.14	.43**	–	
12. Prosociality (SVO angle)	24.03	14.05	.02	−.26**	−.05	.01	−.12	.18*	−.26**	.39**	.05	−.26**	−.38**	–

*Note*: *N* = 174. Punishment severity refers to the punishment received by punished transgressors included in the main analyses. The prosocial, competitive and individualistic attribution scales were constructed based on the pre‐registered exploratory factor analysis. The subdimensions of individualistic attributions (reputation concerns and power‐restoration/self‐esteem) were constructed additionally on theoretical grounds and therefore partially share items with the overall individualistic attribution scale. This conceptual relationship should be considered when interpreting the correlations among these scales. Cronbach's *α* coefficients are reported on the diagonal.

Abbreviations: *M*, mean; *SD*, standard deviation.

* *p* < .05.

** *p* < .01.

The power‐restoration/self‐esteem subdimension of individualistic motive attribution showed a pattern closely mirroring the overall individualistic attribution scale: It was moderately negatively correlated with positive affect and willingness to reconcile and positively correlated with negative affect. It was, however, not significantly related to prosocial behaviour. In contrast, the reputational concerns subdimension only showed small and mostly non‐significant correlations with any of the outcome variables (except for a weak positive correlation with willingness to reconcile).

### Predicting motive attributions (H1‐3)

For all tests reported below, continuous variables were standardized before analyses. We tested Hypotheses 1–3 using pre‐registered linear regression models predicting each motive‐attribution dimension from punishment condition (0 = second‐party punishment, 1 = third‐party punishment), amount stolen, punishment severity and the amount stolen × punishment severity interaction. The same model specification was applied to all five attribution outcomes (prosocial, competitive and overall individualistic motive attribution as derived from the EFA, and both power‐restoration/self‐esteem and reputational concerns based on theoretical considerations). Complete model estimates are summarized in Table 2.

**TABLE 2:** Regression results for prosocial, competitive and individualistic (overall + subdimensions) motive attributions.

Predictor	Prosocial			Competitive			Individualistic			Power/self‐esteem (individualistic)			Reputation (individualistic)		
	*b*	*SE*	*p*	*b*	*SE*	*p*	*b*	*SE*	*p*	*b*	*SE*	*p*	*b*	*SE*	*p*
Intercept	−0.11	0.11	.325	0.05	0.09	.599	−0.09	0.11	.420	0.04	0.10	.709	−0.29	0.11	.008
Condition (0 = 2PP, 1 = 3PP)	0.15	0.16	.353	−0.01	0.12	.942	0.21	0.15	.154	−0.01	0.14	.931	0.45	0.15	.003
Amount stolen	−0.08	0.09	.352	−0.02	0.07	.747	−0.17	0.08	.048	−0.12	0.08	.158	−0.08	0.08	.320
Punishment severity	0.06	0.09	.523	0.64	0.07	< .001	0.37	0.08	< .001	0.44	0.08	< .001	−0.00	0.08	.988
Amount stolen × Punishment severity	0.11	0.09	.226	−0.10	0.07	.151	−0.02	0.08	.798	−0.08	0.08	.340	0.20	0.08	.019
*R* ^2^	.020			.389			.106			.081			.161		

*Note*: *N* = 174. The prosocial, competitive and overall individualistic motive‐attribution scales were constructed based on a pre‐registered exploratory factor analysis. The two individualistic subdimensions (power‐restoration/self‐esteem and reputational concerns) were constructed additionally on theoretical grounds and therefore partially share items with the overall individualistic scale. Condition was coded as 0 = second‐party punishment and 1 = third‐party punishment. Continuous variables were standardized before analysis.

#### Prosocial motive attribution (H1)

Contrary to predictions, participants in the third‐party punishment condition did not attribute punishment more strongly to prosocial motives than participants in the second‐party punishment condition. Likewise, none of the other variables or the interaction term were significant predictors.

#### Competitive motive attribution (H2)

Again, contrary to our hypothesis, second‐ versus third‐party punishment did not significantly predict competitive motive attribution. The only significant predictor in the model was punishment severity, which was positively associated with competitive motive attribution.

#### Individualistic motive attribution (H3)

##### Overall individualistic attribution

The analysis of the overall individualistic attribution scale showed no significant effect of punishment condition, contrary to our hypothesis. Punishment severity was positively associated with individualistic attribution, whereas amount stolen was negatively associated with individualistic attribution. The amount stolen × punishment severity interaction was non‐significant.

##### Power‐restoration/self‐esteem subdimension of individualistic attribution (H3a)

Consistent with the overall individualistic scale, the power‐restoration/self‐esteem subdimension was not predicted by punishment condition. Again, the only significant predictor in the model was punishment severity, which was positively associated with this subdimension of individualistic motive attribution.

##### Reputational concerns subdimension of individualistic attribution (H3b)

In line with our hypothesis, the punishment condition showed a significant effect on reputational attribution. Participants attributed punishment more strongly to reputational concerns when they were punished by a third party compared to a second party. Moreover, the amount stolen × punishment severity interaction was significant. The positive interaction indicates that the positive association between punishment severity and reputational attribution became stronger as the amount stolen increased. All other predictors were non‐significant.

### Predicting downstream consequences (H4)

We tested Hypotheses 4.1–4.4 using linear regression models predicting each downstream outcome (i.e. willingness to reconcile, positive affect, negative affect, prosocial behaviour towards an unrelated person as measured using the SVO) by jointly including the three motive‐attribution scales (as derived from the EFA) as predictors. In addition to these hypothesis tests based on the EFA results, we report results of exploratory follow‐up analyses that regress the outcomes on the theoretically derived motive‐attribution scales (i.e. without excluding any items based on the EFA), including the two subdimensions of individualistic motive attribution (power‐restoration/self‐esteem and reputation concerns). Again, for all tests reported below, continuous variables were standardized before analyses. Complete model estimates are summarized in Table 3.

**TABLE 3:** Regression results for willingness to reconcile, positive and negative affect and prosocial behaviour as predicted by motive attributions.

Predictor	Willingness to reconcile			Positive affect			Negative affect			Prosocial behaviour		
	*b*	*SE*	*p*	*b*	*SE*	*p*	*b*	*SE*	*p*	*b*	*SE*	*p*
Prosocial attribution	0.26	0.08	<.001	0.13	0.07	.071	−0.09	0.08	.270	0.03	0.08	.705
Competitive attribution	−0.46	0.07	<.001	−0.57	0.07	<.001	0.44	0.07	<.001	−0.27	0.08	<.001
Individualistic attribution	−0.07	0.08	.358	−0.11	0.07	.126	0.11	0.08	.170	0.03	0.09	.706
*R* ^*2*^	.258			.373			.229			.070		
Prosocial attribution	0.20	0.08	.012	0.06	0.07	.436	−0.03	0.08	.718	0.10	0.09	.255
Competitive attribution	−0.36	0.08	<.001	−0.51	0.07	<.001	0.38	0.08	<.001	−0.26	0.09	.003
Ind. reputation attribution	0.18	0.09	.038	0.18	0.08	.025	−0.10	0.09	.282	−0.04	0.10	.703
Ind. power/self‐esteem Attr.	−0.21	0.09	.016	−0.21	0.08	.010	0.19	0.09	.035	0.02	0.10	.836
*R* ^*2*^	.304			.403			.242			.076		

*Note*: *N* = 174. Continuous variables were standardized before analysis. The upper panel reports models using the prosocial, competitive and overall individualistic motive‐attribution scales derived from the pre‐registered exploratory factor analysis (EFA). The lower panel reports exploratory follow‐up models using theoretically derived motive‐attribution scales, including prosocial and competitive motive attributions as well as the two individualistic subdimensions: reputational concerns and power‐restoration/self‐esteem concerns. In the lower‐panel models, scales were computed according to theoretical item assignments rather than EFA‐based item exclusions.

#### Willingness to reconcile (H4.1)

In line with our hypotheses, prosocial motive attribution was positively and competitive motive attribution was negatively associated with willingness to reconcile. Contrary to our predictions, individualistic motive attribution was not significantly associated with willingness to reconcile. An exploratory follow‐up analysis using the four theoretically derived motive‐attribution dimensions (including power‐restoration/self‐esteem and reputation concerns as two subdimensions of individualistic motives) revealed that, although the overall individualistic scale was not significantly related to willingness to reconcile, reputational attribution was positively and power‐restoration/self‐esteem attribution was negatively related to willingness to reconcile.

#### Positive affect (H4.2)

Contrary to predictions, neither prosocial nor individualistic motive attribution was significantly related to positive affect. By contrast, competitive motive attribution showed the predicted negative association. Exploratory analyses including the two individualistic subdimensions again revealed diverging patterns: reputational attribution was positively and power‐restoration/self‐esteem attribution was negatively associated with positive affect.

#### Negative affect (H4.3)

Again, neither prosocial nor individualistic motive attribution was significantly related to negative affect, contrasting our hypotheses. By contrast, competitive motive attribution showed the predicted positive association. Exploratory analyses showed that power‐restoration/self‐esteem attribution was positively related to negative affect, whereas reputational attribution was not significantly associated with negative affect.

#### Prosocial behaviour (H4.4)

Again contrasting our hypotheses, neither prosocial nor individualistic motive attribution was significantly associated with prosocial behaviour. By contrast, in line with predictions, competitive motive attribution showed a significant negative association with prosocial behaviour. Exploratory analyses including the two subdimensions of individualistic motive attribution found no significant relations of reputational or power‐restoration/self‐esteem attribution to prosocial behaviour.

### Summary of hypotheses tests

Taken together, the pre‐registered hypotheses tests revealed that whether punishment was administered by a second or third party (i.e. punishment condition) had little systematic influence on transgressors' motive attributions, even though the pre‐registered sample size estimations suggested a small‐to‐medium‐sized effect could be detected with a power ≥ .90. One exception was that third‐party punishment was more strongly attributed to reputational concerns than second‐party punishment. By contrast, punishment severity showed consistent positive associations with competitive, overall individualistic and power‐restoration/self‐esteem‐related attributions. Across downstream outcomes, competitive motive attribution was the most robust correlate of transgressors' affective, relational and behavioural responses, whereas prosocial and overall individualistic motive attributions showed weaker or less consistent associations.

### Additional exploratory analyses

In addition to the pre‐registered analyses, we explored whether punishment condition (0 = second‐party, 1 = third‐party), amount stolen, punishment severity and the amount stolen × punishment severity interaction predicted downstream outcome variables directly. Again, continuous variables were standardized before analyses. Because punishment condition showed little systematic association with motive attributions, except for reputational concerns, any direct effects of condition on downstream outcomes would suggest mechanisms not captured by the measured motive attributions. Complete model estimates are summarized in Table 4.

**TABLE 4:** Regression results for willingness to reconcile, positive and negative affect and prosocial behaviour as predicted by condition (i.e. punisher role), amount stolen, punishment severity and the amount stolen × punishment severity interaction.

Predictor	Willingness to reconcile			Positive affect			Negative affect			Prosocial behaviour		
	*b*	*SE*	*p*	*b*	*SE*	*p*	*b*	*SE*	*p*	*b*	*SE*	*p*
Intercept	0.03	0.10	.789	0.01	0.09	.952	−0.07	0.10	.512	0.14	0.10	.161
Condition (0 = 2PP, 1 = 3PP)	−0.16	0.14	.276	−0.09	0.12	.470	0.34	0.14	.018	−0.40	0.14	.005
Amount stolen	0.02	0.08	.780	0.10	0.07	.127	−0.08	0.08	.338	−0.28	0.08	<.001
Punishment severity	−0.45	0.08	<.001	−0.71	0.07	<.001	0.46	0.08	<.001	−0.16	0.08	.042
Amount stolen × Punishment severity	0.10	0.08	.214	0.08	0.07	.241	−0.20	0.08	.013	0.04	0.08	.576
*R* ^*2*^	.187			.434			.209			.200		

*Note*: *N* = 174. Condition was coded as 0 = second‐party punishment and 1 = third‐party punishment. Continuous variables were standardized before analysis.

Punishment condition significantly predicted negative affect and prosocial behaviour (but not willingness to reconcile or positive affect), with third‐party punishment being associated with higher negative affect and lower prosocial behaviour towards unrelated others. By contrast, punishment severity showed more consistent associations: It was positively associated with negative affect and negatively associated with willingness to reconcile, positive affect and prosocial behaviour. In addition, amount stolen was negatively associated with prosocial behaviour. The amount stolen × punishment severity interaction was negatively associated with negative affect, indicating that the association between punishment severity and negative affect was stronger the fewer points participants stole.

Finally, we explored participants' perceived immorality of their own stealing behaviour in the Stealing Game. Prosocial motive attribution was positively associated with perceived immorality, *b* = 0.31, *SE* = 0.08, *t*(169) = 3.76, *p* < .001, 95% CI [0.15, 0.48], whereas competitive, overall individualistic and both individualistic subdimensions were not significantly related, all *p*s > .27. In a separate model with punishment condition, amount stolen, punishment severity and the amount stolen × punishment severity interaction as predictors, only amount stolen was significantly associated with perceived immorality, *b* = 0.19, *SE* = 0.09, *t*(169) = 2.17, *p* = .032, 95% CI [0.02, 0.36], indicating that participants who had stolen more points perceived their own behaviour as more immoral. All other predictors were non‐significant, all *p*s > .14.

## DISCUSSION

The motive‐attribution framework proposes that punishment affects transgressors not merely through its behavioural consequences but through the motives they attribute to the punisher (Gollwitzer & Okimoto, 2021). The present study tested this idea in an incentivized Stealing Game by examining whether transgressors' motive attributions differed depending on whether punishment was administered by their victims (second‐party punishment) or an uninvolved observer (third‐party punishment), and whether these attributions were associated with affective, relational and behavioural responses. Across analyses, three findings stood out. First, punishment source (i.e. whether punishment was administered by a second or third party) had little systematic influence on motive attributions, with the exception that third‐party punishment was more strongly attributed to reputational concerns. Second, punishment severity was more consistently associated with motive attributions than punishment source. Third, competitive motive attribution was the most robust correlate of downstream consequences of punishment.

### Motive attributions as a function of punishment source

Across analyses, we found little evidence that transgressors systematically differentiated between second‐ and third‐party punishment in their motive attributions. Except for reputational concerns, punishment source did not predict prosocial, competitive, overall individualistic or power‐restoration/self‐esteem attributions. Given the registered‐report format of this research and the adequate power of statistical analyses, these null effects are theoretically informative: They suggest that, at least in anonymous, one‐shot interactions with limited relational context, transgressors may rely less on the role of the punisher in the initial conflict than assumed when interpreting why they were punished. This finding extends previous research that found, for example, that *observers* evaluate second‐ and third‐party punishers differently. According to this research, observers tend to perceive third‐party punishers as more trustworthy and likable than non‐punishing third parties, an effect that has not been found for second‐party punishers (Eriksson et al., 2016; Gordon et al., 2014; Rand et al., 2014). Our findings suggest that such differences in observer evaluations do not necessarily translate into corresponding differences in transgressors' motive attributions. Similarly, the lack of effects on competitive motive attribution contrasts with past literature showing that second‐party punishers often are perceived as more spiteful or retaliatory than third parties (Raihani & Bshary, 2015b, 2019).

The only exception concerned reputational attribution, one theoretically derived subdimension of individualistic motives: Transgressors attributed third‐party punishment more strongly to reputational concerns than second‐party punishment. This finding fits prior work showing that third‐party punishment can yield reputational benefits for punishers (Raihani & Bshary, 2015a) and suggests that transgressors may recognize such reputational incentives. However, because this was the only consistent effect of punishment source, it should be interpreted as a qualified exception rather than evidence that the role of the punisher in the initial transgression broadly structures transgressors' motive attributions.

A more consistent pattern emerged for punishment severity. Across analyses, harsher punishment was associated with stronger competitive and individualistic attributions, whereas the severity of the transgression showed weaker and less consistent associations. This suggests that, for transgressors, the intensity of punishment may be a particularly salient cue for inferring why they were punished. Severe punishment may be difficult to interpret as merely norm‐restorative or prosocial; instead, it may more readily signal hostility, self‐serving motives or a desire to make the transgressor suffer. In this sense, the present findings shift attention from who punishes to how severely punishment is imposed.

### Downstream consequences of motive attributions

With regard to downstream consequences of motive attributions, competitive motive attribution emerged as the most robust and consistent predictor across outcomes (even though it was not the most strongly endorsed attribution descriptively). This pattern may reflect the inherently aversive nature of punishment from the transgressor's perspective. Even when punishment could in principle communicate prosocial motives, its immediate experiential meaning is that another person imposed a loss. Under such ambiguity, transgressors may rely on a relatively simple heuristic: harsher punishment may be taken as indicating stronger hostile intent. Competitive inferences may therefore be more salient and psychologically diagnostic than prosocial ones because they directly explain the harm experienced by the transgressor. Consistent with this speculation, transgressors who attributed punishment to competitive motives reported more negative affect, less positive affect, lower willingness to reconcile and lower prosocial behaviour towards an unrelated other. In contrast, prosocial motive attribution showed only weak and inconsistent associations with these outcomes. Likewise, the overall individualistic attribution scale did not predict affective or behavioural responses.

Importantly, differentiating between the two subdimensions of individualistic motives revealed a more nuanced pattern. Power‐restoration/self‐esteem attribution was associated with higher negative affect and lower willingness to reconcile, whereas reputational attribution was positively related to willingness to reconcile and positive affect. This divergence suggests that individualistic motives are not psychologically homogeneous and that power/self‐esteem‐related versus reputation‐related interpretations of punishment may have distinct interpersonal implications. Of note, because this distinction was theoretically derived and not supported by the EFA, these findings should be interpreted cautiously; but they point to potentially meaningful differences within individualistic interpretations of punishment.

Overall, the present findings are broadly consistent with core assumptions of the motive‐attribution framework, which posits that transgressors' motive attributions are important for understanding post‐punishment reactions and conflict resolution (Gollwitzer & Okimoto, 2021). In line with this view, prosocial attribution tended to align with more constructive relational outcomes, whereas competitive attribution was associated with relational deterioration. At the same time, because motive attributions were measured rather than experimentally manipulated, these associations cannot be interpreted causally and may partly reflect stable individual differences in attributional or emotional tendencies. Future research is therefore needed to identify which factors shape transgressors' attributions of punishment and under what conditions such attributions foster more constructive conflict‐related outcomes.

### Limitations and future directions

Several limitations of the present research warrant consideration. First, we employed the Stealing Game as our experimental paradigm, which allowed us to examine motive attributions in response to an intentional and consequential transgression. At the same time, several conceptual trade‐offs of the paradigm should be acknowledged, particularly regarding the generalizability of our findings. Most notably, participants' interactions took place in an anonymous online environment, which may have reduced emotional salience and reputational concerns compared to real‐world or face‐to‐face contexts. Motive attributions may unfold differently in ongoing interpersonal relationships, where social history, group membership or status hierarchies shape interpretations of punishment. Relatedly, prior tests of the motive‐attribution framework have often relied on scenario‐based designs that explicitly embedded punishment in relational contexts (de Vel‐Palumbo et al., 2023; Twardawski et al., 2023). While our design deliberately prioritized internal validity over ecological generalizability, this may have limited the extent to which transgressors attributed punishment to prosocial motives.

Second, although our findings align with key assumptions of the motive‐attribution framework, the present design does not allow for causal inferences regarding the downstream effects of motive attributions. Motive attributions were measured and not experimentally manipulated, making it unclear whether competitive attribution drives negative affect, willingness to reconcile and prosocial behaviour or whether transgressors who react more negatively to punishment are also more prone to infer malevolent motives. Future research could address this limitation by experimentally manipulating how punishment is framed or communicated, thereby directly shaping motive attributions and allowing for stronger causal tests of the framework. Such studies could also test whether competitive interpretations become especially salient under ambiguity, and whether contextual information, time to reflect or explicit communicative cues help transgressors assign more weight to prosocial interpretations.

Third, the study was conducted in a Western, educated, industrialized, rich and democratic (WEIRD) context using a participant pool largely consisting of university students. As a result, the generalizability of the findings to other populations or cultural contexts remains an open question. This limitation is particularly relevant given substantial cross‐cultural variation in how norm violations are interpreted and how punishment is used and evaluated across societies (Molho et al., 2024).

### CONCLUSION

Overall, the present findings suggest that transgressors' interpretations of punishment were less clearly shaped by *who* punished them and more closely linked to the severity of the punishment they received. Contrary to expectations, transgressors did not systematically attribute different motives to second‐ and third‐party punishers, with the exception that reputational concerns were more strongly attributed to third‐party punishers. Instead, harsher sanctions were associated with stronger competitive and individualistic motive attributions. Moreover, competitive motive attribution was associated with increased negative affect, but reduced willingness to reconcile and prosocial behaviour. Together, these findings underscore the importance of considering punishment severity and transgressors' motive attributions for understanding post‐conflict dynamics. Punishment may function as a social signal to transgressors. The interpretation of that signal, however, appears to be more closely linked to the severity with which punishment is imposed than to the punisher's role in the initial transgression.

## AUTHOR CONTRIBUTIONS

**Mathias Twardawski:** Conceptualization; methodology; data curation; investigation; validation; formal analysis; supervision; funding acquisition; visualization; project administration; writing – original draft; writing – review and editing. **Dorothee Mischkowski:** Conceptualization; methodology; data curation; investigation; validation; supervision; funding acquisition; project administration; writing – original draft; writing – review and editing. **Stefanie Hechler:** Conceptualization; methodology; data curation; investigation; validation; supervision; writing – original draft; writing – review and editing.

## CONFLICT OF INTEREST STATEMENT

The authors declare that they have no conflicts of interest.

## Data Availability

The pre‐registration, materials, data, codebook and analysis script of the study are available on the Open Science Framework: https://osf.io/w7abx.

## Footnotes

^1^
Note that the motive‐attribution framework hypothesizes that transgressors attribute these motives independently from each other (Gollwitzer & Okimoto, 2021), although correlations may be expected depending on the situation. Specifically, competitive and individualistic motive attributions have correlated positively with each other in the past; whereas both correlated negatively with the attribution to prosocial motives (de Vel‐Palumbo et al., 2023; Twardawski et al., 2023).

^2^
These items were designed to target the most critical features of the paradigm (e.g. how many points can be stolen). They were intended to ensure that participants fully understand the core structure of the experimental task, particularly the roles of the different persons involved and the potential consequences of their actions. This comprehension is essential for meaningful interpretation of their behaviour and responses.

^3^
To obtain real interactions, we also collected data from participants assigned to the roles of Person B and Person C. To increase efficiency in data collection, participants assigned to these roles made decisions in both roles (i.e. for their own interaction as Person B, as well as for a different interaction as Person C). That is, participants not assigned to the role of Person A took on the roles of both Person B and Person C, one after another and made punishment decisions for two separate interactions with two different Persons A. Specifically, during the time Person A made their stealing decision, participants in the roles of Person B/C indicated their punishment responses using the strategy method (i.e. making conditional decisions for each possible stealing amount; Brandts & Charness, 2011; Selten, 1967). Importantly, their behaviour in the game was also incentivized, as outcomes depended on their own decisions and the corresponding decisions of the relevant Person A. For payment of participants in the roles of Person B/C, the outcome for Person B in their interaction with Person A was always relevant (= initial endowment of Person B minus stealing by Person A). The further payment of Person B/C depended on whether stealing was detected and if so, for which role: In 10% of cases (non‐detection of stealing), Person B's outcome after stealing determined payment for participants in the roles of Person B/C (i.e. they did not receive additional endowment to punish). When Person A's decision was detected by Person B (in 45% of cases), the participant's punishment decision as Person B was additionally relevant, depending on Person A's behaviour in their interaction (as determined via the strategy method). When Person A's decision was detected by Person C (in the remaining 45% of cases), the participant's punishment decision as Person C was additionally relevant for their payment, based on Person A's behaviour in the other interaction (again, as determined via the strategy method). Participants in the roles of Person B/C also completed several questionnaires for exploratory purposes (e.g. on their actual punishment motives; not outlined in the present manuscript but detailed in the materials on OSF) and responded to the SVO Slider Measure. Ultimately, we recruited participants in the roles of Person B and C until the planned sample size for the punished transgressor (i.e. Person A, see Sample section) had been reached.

^4^
Note that personal data (e.g. name and bank account number) were stored separately to assure anonymity within the experiment.

^5^
Again, note that the three broad motive‐attribution scales (prosocial, competitive and individualistic) were derived from the pre‐registered EFA, whereas the two subdimensions of individualistic attributions (power‐restoration/self‐esteem and reputational concerns) were additionally constructed on theoretical grounds. As a consequence, the subdimensions partially rely on overlapping item sets with the overall individualistic attribution scale, which should be considered when interpreting their intercorrelations.
